# Geometrical Custom Modeling of Human Cornea In Vivo and Its Use for the Diagnosis of Corneal Ectasia

**DOI:** 10.1371/journal.pone.0110249

**Published:** 2014-10-17

**Authors:** Francisco Cavas-Martínez, Daniel G. Fernández-Pacheco, Ernesto De la Cruz-Sánchez, José Nieto Martínez, Francisco J. Fernández Cañavate, Alfredo Vega-Estrada, Ana B. Plaza-Puche, Jorge L. Alió

**Affiliations:** 1 Department of Graphical Expression, Technical University of Cartagena, Cartagena, Spain; 2 Department of Physical Activity and Sport, University of Murcia, Murcia, Spain; 3 Vissum Corporación Oftalmológica, Alicante, Spain; Cedars-Sinai Medical Center; UCLA School of Medicine, United States of America

## Abstract

**Aim:**

To establish a new procedure for 3D geometric reconstruction of the human cornea to obtain a solid model that represents a personalized and in vivo morphology of both the anterior and posterior corneal surfaces. This model is later analyzed to obtain geometric variables enabling the characterization of the corneal geometry and establishing a new clinical diagnostic criterion in order to distinguish between healthy corneas and corneas with keratoconus.

**Method:**

The method for the geometric reconstruction of the cornea consists of the following steps: capture and preprocessing of the spatial point clouds provided by the Sirius topographer that represent both anterior and posterior corneal surfaces, reconstruction of the corneal geometric surfaces and generation of the solid model. Later, geometric variables are extracted from the model obtained and statistically analyzed to detect deformations of the cornea.

**Results:**

The variables that achieved the best results in the diagnosis of keratoconus were anterior corneal surface area (ROC area: 0.847, p<0.000, std. error: 0.038, 95% CI: 0.777 to 0.925), posterior corneal surface area (ROC area: 0.807, p<0.000, std. error: 0.042, 95% CI: 0,726 to 0,889), anterior apex deviation (ROC area: 0.735, p<0.000, std. error: 0.053, 95% CI: 0.630 to 0.840) and posterior apex deviation (ROC area: 0.891, p<0.000, std. error: 0.039, 95% CI: 0.8146 to 0.9672).

**Conclusion:**

Geometric modeling enables accurate characterization of the human cornea. Also, from a clinical point of view, the procedure described has established a new approach for the study of eye-related diseases.

## Introduction

Characterization of corneal topography is critical for the assessment of vision quality and for several clinical applications including the diagnosis and management of corneal diseases [1]–[3], the planning of refractive surgery [4]–[5], and the construction of corneal numerical models [6].

At present, several non-invasive technologies that do not require the use of anesthesia or contact with the cornea have been developed for the comprehensive characterization of corneal topography. These include Scheimpflug photography [7], a combination of scanning-slit and Placido-disc technologies [8], very-high-frequency ultrasonography [9], and optical coherence tomography [10]–[12]. Scheimpflug photography–based systems allow the study and characterization of both anterior and posterior corneal surfaces [13]–[14]. Different studies have validated the consistency of the measurements obtained with this technique, using different commercially available devices [15]–[16]. The combination of accurate Scheimpflug photography analysis for corneal characterization with classical Placido-disc technology [17] has been recently developed, with the aim of maintaining the benefits of the Scheimpflug technology and optimizing the measurements of the anterior corneal curvature. This combined technology has been shown to provide highly consistent anterior and posterior corneal curvature measurements [18]–[20]. These data allow the human cornea to be modeled in order to detect corneal ectatic disorders, such as keratoconus.

Modeling of the human cornea can be approached by two different strategies: i) using a generic model which is valid to reproduce and extract results that can be applied to the whole population, or ii) creating a personalized model that allows the particular case of a specific patient to be studied. Both types of models have been reported in the literature [21]–[22] using a corneal geometry approach consisting of a base or regular surface (B) and a residue (R), which represents the local and global irregularities of the corneal topography with respect to the regular base model (B). Other authors simulate the human cornea using finite-elements models [23]–[27]. However, the reconstructions performed until now have the following problems:

- The low density of data from the posterior corneal surface provided by the ophthalmological devices makes it difficult to obtain a full 3D reconstruction of the human cornea [28]–[29].
- Most of the mathematical models used in the approaches are based on the Zernike polynomial, which has problems that have been widely discussed in the literature [30]–[32]. Some authors try to solve this issue using first a coarse adjustment by means of the Zernike polynomial and secondly a fine adjustment based on a lineal combination of radial basis Gaussian functions [33]. However, this method does not properly represent the corneal geometry when it has high irregularity levels due to corneal ectatic disorders, both on anterior and posterior surfaces.

For all the above-mentioned reasons, this paper proposes a procedure for the geometric reconstruction of the human cornea which allows a 3D solid model to be obtained which reproduces the actual and personalized morphology of both the anterior and posterior surfaces of the cornea. Once the 3D model has been created, several geometric variables are defined from the model, obtaining a personalized characterization of the corneal topography. The study of these variables allows a new clinical diagnostic criterion to be established which enables healthy corneas to be distinguished from corneas with keratoconus, in which the deformation of the cornea directly affects several of the geometric variables studied.

## Methods

### Participants

A total of 131 subjects (aged 36.03±13.41 years old) volunteered to participate in this study. Participants were recruited in Vissum Alicante (Vissum Corporation, Alicante, Spain). The subjects were informed in detail about the procedures.

All subjects were divided into two different groups according to the presence or absence of keratoconus:

1. The first group did not present any ocular pathology and consisted of 90 healthy eyes of 90 patients with an age range of between 7 and 66 years old. Participants with any ocular or corneal pathology, or those whose eyes had undergone any previous procedure, were excluded.
2. The second group of eyes with ocular pathology consisted of 41 clinically diagnosed keratoconic eyes of 41 patients, with an age range of between 14 and 65. Rabinowitz criterion was used for clinical diagnosis of keratoconus, which considers the presence of a localized corneal topography curving and/or the presence of an asymmetric bow tie with or without topographical angulated principal meridians. Moreover, we also considered any microscopic keratoconic sign [34]–[36]: stromal thinning, Fleischer ring, Vogt striae, anterior corneal scars in the corneal stroma or protrusion apex. Exclusion for this group was considered when there was any previous eye surgery or any other eye disease. The severity of the disease in this group was rated according to the Amsler-Krumeich classification [36]–[38].

Only one eye per patient was included in both groups, following a numerical sequence (dichotomous sequence 0 and 1) created by computer software in order to avoid interference potential correlations that could exist between the eyes of the same person.

This study was approved by the Vissum Corporacion Oftalmologica’s Clinical Research Ethics Committee, and was performed in accordance with the ethical standards laid down in the 1964 Declaration of Helsinki. Written informed consent was obtained after explaining the nature of the procedure prior to surgery in all cases.

### Measurement protocol

#### Eye exam

All eyes selected underwent a thorough and comprehensive eye and vision examination which included uncorrected distance visual acuity (UDVA), corrected distance visual acuity (CDVA), manifest refraction, Goldmann tonometry, biometry (IOLMaster, Carl Zeiss Meditec AG) and corneal topographic analysis with Sirius System (CSO, Florence, Italy). All measurements were performed by the same experienced examiner. With respect to the corneal topographic analysis, three consecutive measurements were performed to calculate the average values for posterior analysis.

The Sirius topographer is an ophthalmic instrument that uses a rotating Scheimpflug camera [39] with a Placido disc [14] to obtain the corneal topography. In a few seconds it captures and processes 25 image sections made with the Scheimpflug camera and the image of 22 rings of a Placido disc projected onto the cornea. The device has a second chamber to control the correct alignment of the system with the eye for data acquisition. The corneal topographer has a good level of consistency for taking measurements [16] of sagital and tangential curvature of both faces of the corneal refractive power, points of the anterior and posterior corneal surface, corneal pachymetry and estimations of other biometric structures above, such as the anterior chamber depth segment. Data registration for the present study was performed with the Phoenix (Phoenix, CSO, Florence, Italy) software.

#### Geometrical modeling

The method proposed in this article for the geometric reconstruction of the cornea consists of the following steps:

*i) Preparation of the point cloud.* The proposed reconstruction process is based on the generation of a surface from the geometry that a point cloud presents in a coordinate system for a three-dimensional space, usually in Cartesian coordinate format. This technique of geometric reconstruction is not new in the field of biomedical engineering, having already been successfully used in the reconstruction of other parts of the body [40]–[41].

The point cloud reconstruction of the geometry of the anterior and posterior surfaces of the cornea was obtained by the Sirius corneal topographer. However, this device has a low density of data in the geographical area of the cornea known as the ‘peripheral zone’ (radius 4 mm to 5.5 mm) and in the limbo (6 mm radius), due to the time taken for rotation and data collection. This suboptimal performance of the device is caused by the presence of intrinsic patient factors in the measurement process, such as the stability of the tear film, or an obstruction of the visual field by tabs or inadequate eyelid opening at the moment of the data collection. This led to the development of a method for geometric reconstruction that comprises the corneal surface from its geometric center (r = 0 mm) to the beginning of the so-called peripheral zone (r = 4 mm), which is mainly justified by the following two reasons:

- Geometric principle. The Sirius corneal topographer permits the collection of all the points that make up the geometry of the cornea in the region defined for reconstruction (r = 0 to 4 mm). Specifically, 10752 spatial points corresponding to both anterior and posterior corneal surfaces (5376 for each one) were obtained for each patient of the population under study, including both healthy and diseased cases.
- Clinical principle. The corneal surface defined for the study (r = 0 to 4 mm) is considered to have more information on corneal morphology for both healthy and diseased eyes. This region includes both the central area (r = 0 to 2 mm), which corresponds to the more spherical area with more visual impact and accounts for 25% of keratoconus cases, and the paracentral area (r = 2 to 4 mm), which corresponds to the area where the cornea begins to flatten and accounts for 72% of keratoconus cases [42]. In total, the region targeted for the geometric reconstruction (r = 0 to 4 mm) presents levels of irregularity in corneal morphology for both anterior and posterior surfaces which encompasses 97% of keratoconus cases.

The Sirius corneal topographer can provide raw data of the spatial points that conform both anterior and posterior corneal surfaces, indicating the coordinates (X, Y, Z) of every scanned point. This data is the most reliable information to be used due to it has not been processed by any software algorithm or manipulated [17]. For this reason the raw data provided by the topographer was used in this study.

**Figure 1:**
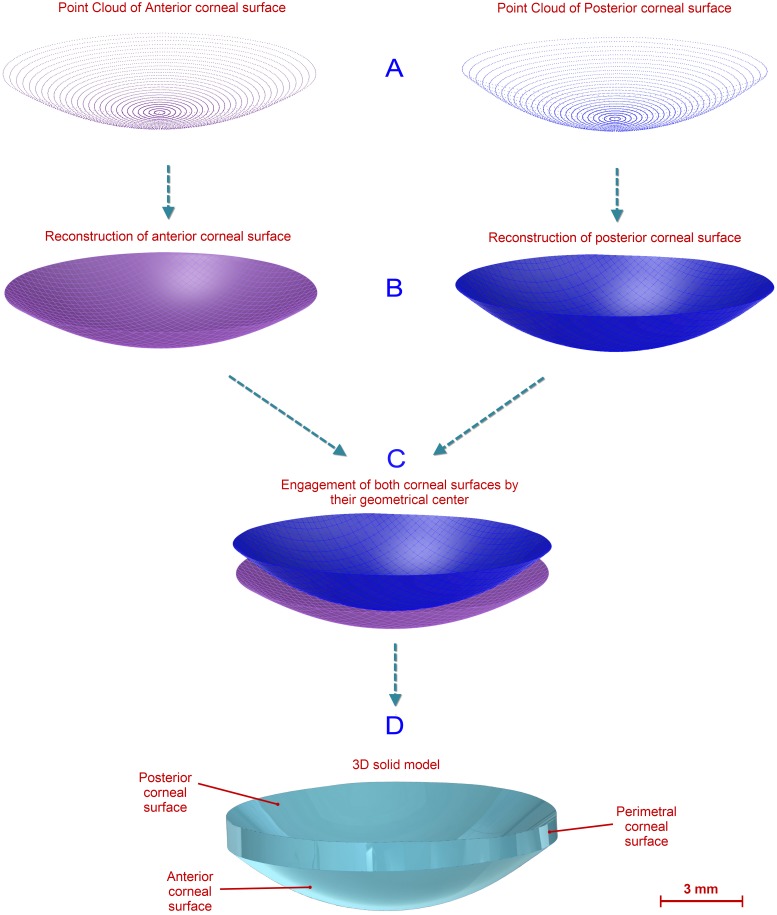
Scheme of the 3D geometric reconstruction procedure of the cornea.

Due to the Sirius topographer only providing spatial points data in Cartesian format for the anterior corneal surface (not for the posterior surface), it was necessary to export data in polar format to obtain data from both corneal surfaces. This data was given as a CSV table where every row represented a circle in the map and every column represented a semi-meridian, giving 256 points for each radius. This way, each i-th row sampled a map on a circle of i*0.2 mm radius, and each j-th column sampled a map on a semi-meridian in the direction of j*360/256°, so each Z value of the matrix [i, j] represented the point P (i*0.2, j*360/256°) in polar coordinates. In order to perform these calculations, exported data were further formatted in Cartesian coordinates by an algorithm programmed using Matlab software (Fig. 1-A).

*ii) Geometric Surface Reconstruction.* The point cloud representing the corneal geometry was imported by the surface reconstruction software Rhinoceros v5.0. This uses a mathematical model to generate surfaces based on non-uniform rational B-spline (NURBS) [43]. The surface generated from these spatial points is characterized by two parametric directions *u* and *v*. Besides, these types of surface are invariant under affine or perspective transformations, providing the flexibility to design a wide variety of surfaces with low memory consumption when compared to other methods.

**Figure 2:**
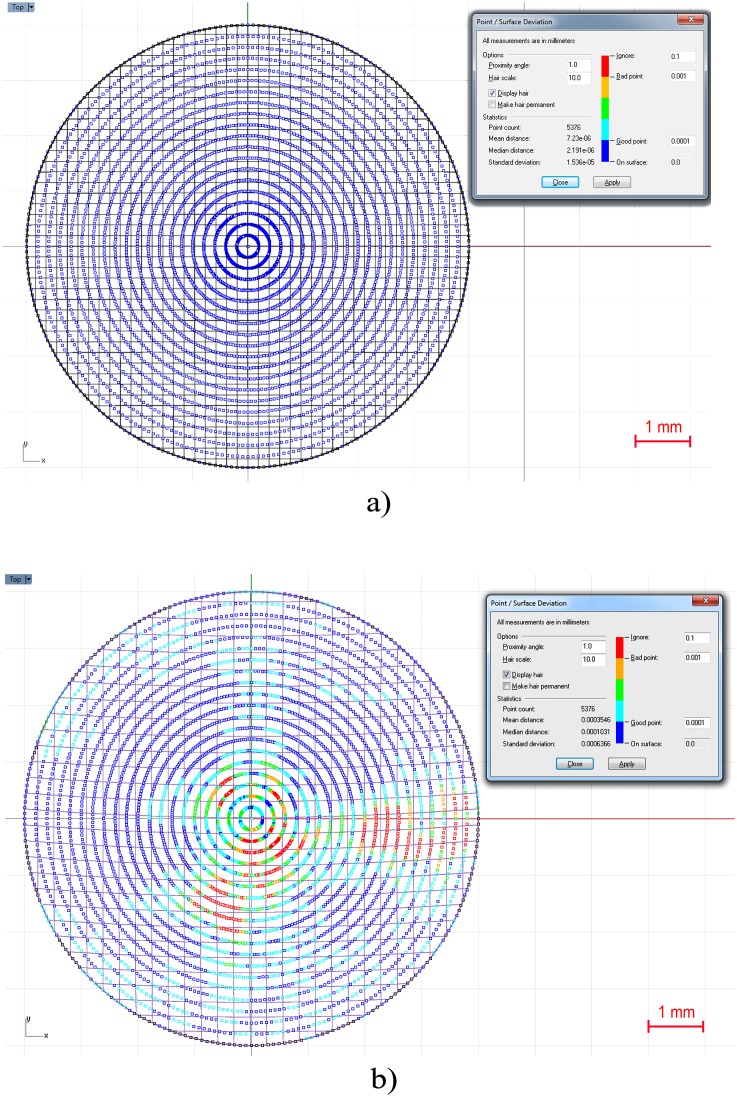
Analysis of the point-surface deviation for the anterior surface reconstruction of: a) a healthy cornea, b) a cornea with advanced keratoconus.

The surface that best fits the point cloud was generated with the Rhinoceros’s patch surface function (Fig. 1-B), a reconstruction software option that fits a surface through given curves, meshes, point objects, and point clouds [44]. For this research, this function tried to minimize the nominal distance between the 3D point cloud and the solution surface. For this objective, the function was configured by setting the sample point spacing at 256 (number of points for each data ring), the surface span planes at 255 for both u and v directions (the maximum number of span planes that the software permitted), and the stiffness of the solution surface at 10^−3^ [mm]. This last parameter provides information on how much the best fit plane can be deformed in order to match the input points. This deviation can be calculated later by the software, providing a mean value of the distance error for the solution surface. This can be seen in Figure 2a, where the top view of the point cloud for the anterior surface of a healthy cornea is represented and a mean distance error of 7.23×10^−6^±1.536×10^−5^ [mm] (mean ± standard deviation) is obtained. Figure 2b shows the deviation error for the anterior surface of a cornea with advanced keratoconus, obtaining in this case a mean distance error of 3.54×10^−4^±6.36×10^−4^ [mm] (mean ± standard deviation). In both figures the same good/bad threshold values have been configured: 10^−3^ [mm] for bad points (in red) and 10^−4^ [mm] for good points (in blue). These figures show how the points are distributed in perfect circular rings from r = 0 mm to r = 4 mm in steps of 0.2 mm. This is because, as previously mentioned, the Sirius device gives the 3D points in polar coordinates, and once converted into Cartesian format, they are distributed in a circular map.

Using this procedure, anterior and posterior cornea surfaces with radius 4 mm were generated, and engaged by their geometrical center and Z axis (Fig. 1-C). Both surfaces and the perimetric surface (bonding surface between both sides in the Z-axis direction) were then joined to form a single surface.

**Figure 3:**
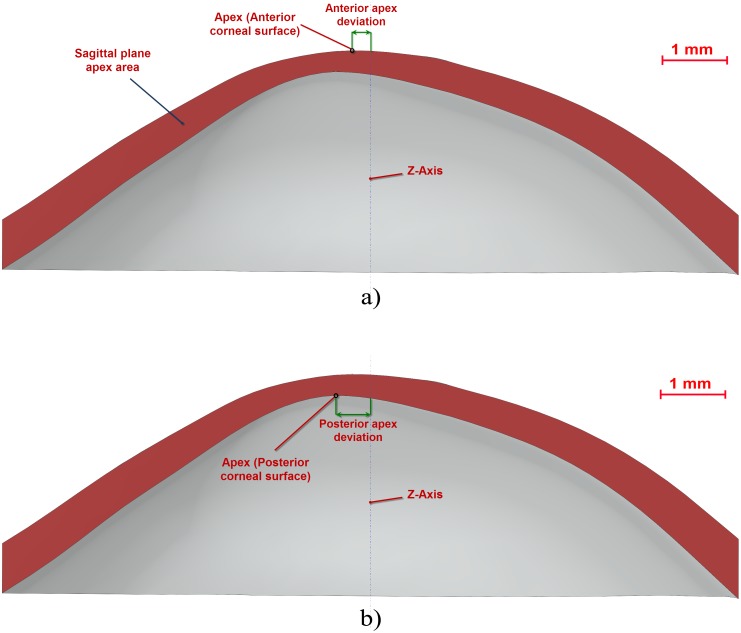
Sagittal plane of the cornea: a) passing through anterior apex and the Z-axis, b) passing through posterior apex and the Z-axis.

**Figure 4:**
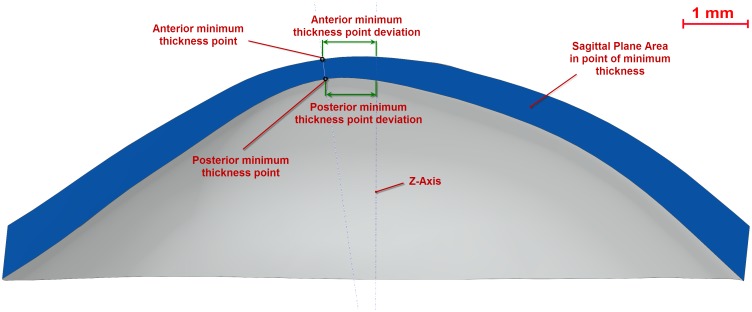
Sagittal plane of the cornea passing through the Z axis and minimum thickness points of both corneal surfaces.

**Figure 5:**
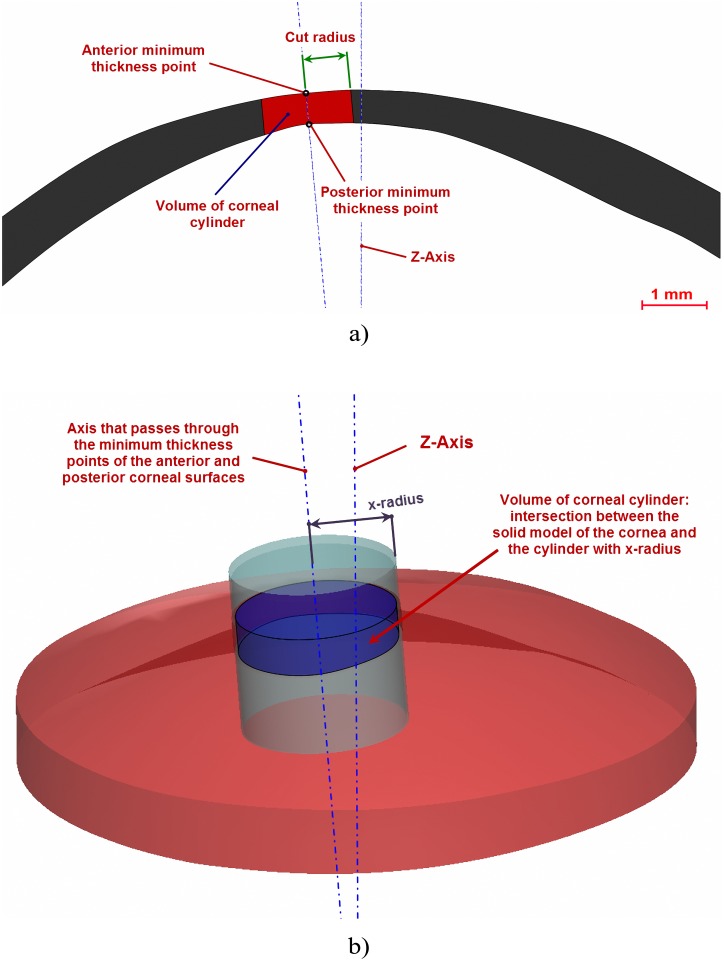
Volume of corneal cylinder with a determined radius: a) 2D view of the cylinder and its parameters, b) 3D view of the intersection between the solid model of the cornea and the cylinder.

*iii) Solid Modeling.* The resulting surface was imported using the solid modeling software SolidWorks v2012, which allowed the generation of the solid model that is representative of the custom and actual geometry of each cornea (Fig. 1-D). Taking into account all previously mentioned aspects about the data to be used for the model reconstruction, it is important to clarify that the solid models were reconstructed up to a 4 mm radius. Any case in which the data provided by the Sirius topographer had some point left until the 4 mm radius was discarded from this study.
*iv) Definition of geometric variables. F*rom the solid model obtained, the following geometric variables were defined:

- Total corneal volume [mm^3^] (Fig. 1-D): volume limited by front, back and peripheral surfaces of the solid model generated.
- Anterior corneal surface area [mm^2^] (Fig. 1-D): area of the front/exterior surface.
- Posterior corneal surface area [mm^2^] (Fig. 1-D): area of the rear/interior surface.
- Total corneal surface area [mm^2^] (Fig. 1-D): sum of anterior, posterior and perimetral corneal surface areas of the solid model generated.
- Sagittal plane apex area [mm^2^] (Fig. 3a): area of the cornea within the sagittal plane passing through the Z axis and the highest point (apex) of the anterior corneal surface.
- Anterior and posterior apex deviation [mm] (Figs. 3a and 3b): average distance from the Z axis to the highest point (apex) of the anterior/posterior corneal surfaces.
- Sagittal plane area in minimum thickness points (maximum curvature) [mm] (Fig. 4): area of the cornea within the sagittal plane passing through the Z axis and the minimum thickness points (maximum curvature) of the anterior and posterior corneal surfaces.
- Anterior and posterior minimum thickness point deviation (maximum curvature) [mm] (Fig. 4): average distance in the XY plane from the Z axis to the minimum thickness points (maximum curvature) of the anterior/posterior corneal surfaces.
- Center of mass coordinates X, Y, Z of the solid [mm].
- Net Deviation from the center of mass in XY [mm]: projective XY Modulus of the center of mass.
- Volume of corneal cylinder with r-x [mm^3^]: volume of the intersection in 3D (see Fig. 5b) between the solid model of the cornea generated and a cylinder with x radius whose axis passes through the minimum thickness points (maximum curvature) of the anterior and posterior corneal surfaces (see Fig. 5a). Radiuses adopted for this study were 0.5, 1, 1.5 and 2 mm.

### Statistical analysis

Data engagement scores were confirmed by means of the Kolmogorov–Smirnov test. According to this analysis, a Student’s t-test or U-Mann Whitney Wilcoxon test for unpaired data was performed (depending on normality), in order to describe differences between normal and keratoconus groups in all the measurements proposed. An additional Kruskal-Wallis test was used to compare differences between groups according to disease stages (Amsler– Krumeich grading system). Pairwise comparisons were performed using Dunn’s procedure with a Bonferroni correction for multiple comparisons. Finally, a ROC curve analysis was performed in order to obtain the accuracy of the different measurements. An area of 1 represents the most accurate test, while an area of 0.5 represents a worthless test. The closer the curve follows, the left-hand border, and then, the top borders of the ROC space, the more accurate the test is. So this is an estimation of the ability of these measurements to identify more true positives while minimizing the number of false positives. All the statistical analyses mentioned were performed using Graphpad Prism 6 and SPSS 17.0 software.

## Results

From the total of 131 patients, this study included a total of 90 healthy eyes (persons aged from 7 to 66 years old) and 41 eyes (persons aged from 14 to 66 years old) with keratoconus diagnosis in several grades (51.2% in stage I, 36.6% in stage II, 12.2% in the most extreme stages, III and IV).

Most of the modeled variables showed differences between normal and keratoconus diagnosed eyes, as seen in table 1: total corneal volume is greater in normal eyes (p<0.000), anterior and posterior corneal surface areas are smaller in the healthy subjects (p<0.000). This pattern of difference can be seen for most of variables studied: normal eyes have a greater sagittal plane apex area and a greater sagittal plane area in minimum thickness points (p<0.000), whereas anterior and posterior apex deviations are greater in keratoconus eyes (p<0.000) and also in anterior and posterior minimum thickness point deviation (p<0.01). The eyes’ center of mass is not a good predictor of differences between normal and keratoconus eyes, and there is no statistical difference in x, y and z, or in net deviation from the center of mass in x, y. Also, the total corneal area modeled does not show differences between groups.

**Table 1:** Descriptive values (mean and 95% CI) and differences between normal and keratoconus corneal variables modeled.

Measurement	Normal Group,n = 90	Keratoconus Group,n = 41	
	Mean (95% CI)	Mean (95% CI)	p (statistical test)
Total corneal volume [mm^3^]	25.81(25.47–26.14)	23.42(22.81–24.03)	0.000(Mann–Whitney)
Anterior corneal surface area[mm^2^]	43.08(43.04–43.11)	43.39(43.30–43.40)	0.000(Mann–Whitney)
Posterior corneal surface area[mm^2^]	44.24(44.18–44.30)	44.73(44.57–44.89)	0.000(Mann–Whitney)
Total corneal surface area [mm^2^]	103.93(103.67–104.20)	103.59(103.13–104.05)	0.169(Mann–Whitney)
Sagittal plane apex area [mm^2^]	4.33(4.27–4.39)	3.90(3.80–4.00)	0.000(Mann–Whitney)
Sagittal Plane Area in minimumthickness points [mm^2^]	4.32(4.26–4.38)	3.88(3.78–3.99)	0.000(t-test)
Anterior apex deviation [mm]	0.0003(0.0001–0.0006)	0.0083(0.0048–0.0118)	0.000(Mann–Whitney)
Posterior apex deviation [mm]	0.0768(0.063–0.0905)	0.1886(0.1587–0.2185)	0.000(Mann–Whitney)
Center of mass X [mm]	0.044(0.0409–0.0478)	0.0415(0.0331–0.0499)	0.341(t-test)
Center of mass Y [mm]	0.034(0.0304–0.0375)	0.0364(0.0279–0.0449)	0.964(Mann–Whitney)
Net deviation from center ofmass XY [mm]	0.0577(0.0538–0.0616)	0.0606(0.0517–0.0694)	0.132(t-test)
Center of mass Z [mm]	0.771(0.766–0.776)	0.785(0.771–0.800)	0.156(Mann–Whitney)
Anterior minimum thicknesspoint deviation [mm]	0.864(0.812–0.917)	1.031(0.901–1.161)	0.010(Mann–Whitney)
Posterior minimun thicknesspoint deviation [mm]	0.800(0.749–0.851)	0.958(0.835–1.081)	0.009(Mann–Whitney)

**Figure 6:**
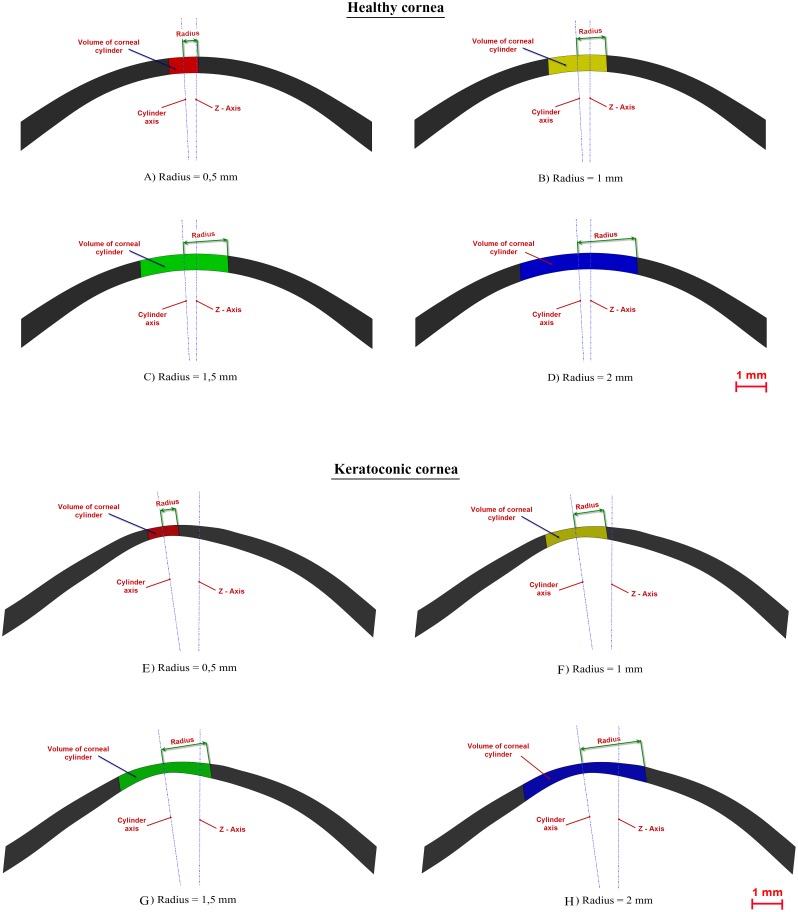
Schematic representation of the difference in the volume of corneal cylinder with radius 0.5, 1.0, 1.5 and 2.0 between a normal cornea (A–D) and a cornea with keratoconus (E–H).

Additionally, figure 6 shows the differences between normal and keratoconus groups in the volumes of corneal cylinder modelled using different radius (maximum bending in 0.5, 1.0, 1.5 and 2.0 mm from the axis that passes through the minimum thickness points). Please note the differences between a normal eye (Figs. 6a–d) versus one with keratoconus (Figs. 6e–h). Statistical differences were found for all these geometric variables, enabling the differentiation of keratoconus eyes (see table 2).

**Table 2:** Differences between the normal group (Figs. 7a–d) versus the keratoconus group (Figs. 7e–h) in the volume of corneal cylinder with radius 0.5, 1.0, 1.5 and 2.0 mm (mean and standard deviation; U-Mann-Whitney-Wilcoxon test).

Volume of corneal cylinder (mm^3^)with Radius×(mm)	Normal Group,n = 90 (figs. 7a–d)	Keratoconus Group,n = 41 (figs. 7e–h)		
	Mean±SD	Mean±SD	z	p
r = 0.5	0.46±0.29	0.35±0.041	–8.280	0.000
r = 1	1.71±0.11	1.46±0.15	–7.980	0.000
r = 1.5	3.91±0.24	3.33±0.50	–7.650	0.000
r = 2	7.10±0.44	6.26±0.57	–7.211	0.000

The outcomes according to keratoconus severity are shown in Table 3, where comparisons are established according to the severity of the disease following the Amsler–Krumeich grading system. Only total corneal surface area and center of mass in x and y are not suitable for differentiating between groups, with the other geometrical variables being statistically significant between stages (from p<0.000 to p<0.013). For center of mass in z and net deviation from the center of mass in xy, it can be observed that III and IV stages are well differentiated from the other groups, as post-hoc analysis reveals (Dunn’s method and Bonferroni correction for multiple comparisons). Note that the calculated effects size for each disease stage (versus normal group) allows quantifying the degree of change, greater for stages III and IV in most of the variables, becoming more evident with the progress of the disease.

**Table 3:** Comparison between groups of the outcomes modeled; Kruskall-Wallis test (with p values) and effect size, (ES)^1^.

	Normal	I Stage	II Stage	III–IV Stage	p (Krskall-Wallis test)
Total cornealvolume [mm^3^]	26.00[21.37–29.50]	23,88[19,82–26.66]	23,32[19,09–27,60]	19,78[16,97–22,60]	0.000
(ES)	-	1,19	1,42	3,35	
Anterior cornealsurface area [mm^2^]	43,07[42,73–43,38]	43,27[42,89–43,58]	43,46[43], [14]–[44], [12]	44,10[43,86–44,35]	0.000
(ES)	-	–1,18	–1,70	–4,85	
Posterior cornealsurface area [mm^2^]	44,25[43,49–44,90]	44,55[43,93–45,07]	44,85[44,21–45,90]	45,75[45,72–45,78]	0.000
(ES)	-	–0,99	–1,54	–4,18	
Total cornealsurface area [mm^2^]	103,94[100,69–106,15]	103.59[100,91–104–75]	103,71[99,97–106,18]	103,58[101,68–105,48]	0.185
(ES)	-	0,28	0,18	0,28	
Sagittal planeapex area [mm^2^]	4,34[3,58–5,00]	3,94[3,28–4,50]	3,91[3,19–4,48]	3,35[3,00–3,70]	0.000
(ES)	-	1,30	1,37	3,25	
Sagittal Plane Area inminimum thicknesspoints [mm^2^]	4,35[3,57–5,01]	3,94[3,28–4,50]	3,92[3,03–4,48]	3,34[2,99–3,69]	0.000
(ES)	-	1,33	1,28	3,29	
Anterior apexdeviation [mm]	0,000[0,000–0,007]	0,003[0,000–0,016]	0,008[0,000–0,041]	0,029[0,014–0,044]	0.000
(ES)	-	–1,12	–1,41	–5,93	
Posterior apexdeviation [mm]	0,068[0,024–0,650]	0,164[0,054–0,339]	0,198[0,032–0,453]	0,132[0,054–0,209]	0.000
(ES)	-	–1,25	–1,44	–0,97	
Center of mass X[mm]	0,044 [0,009–0,089]	0,042[0,002–0,083]	0,030[0,006–0,083]	0,059[0,002–0,116]	0.184
(ES)	-	0,12	0,79	–0,82	
Center of mass Y[mm]	0,032 [0,000–0,095]	0,036[0,005–0,084]	0,021[0,008–0,281]	0,065[0,001–0,129]	0.187
(ES)	-	–0,22	0,37	–1,67	
Net deviation fromcenter of mass XY[mm]	0,056[0,009–0,108]	0,059[0,031–0,105]	0,042[0,014–0,281]	0,123[0,116–0,129]	0.006
(ES)	-	–0,16	0,48	–3,25	
Center of mass Z[mm]	0,770[0,708–0,813]	0,771[0,730–0,795]	0,799[0,709–0,864]	0,839[0,809–0,869]	0.013
(ES)	-	–0,04	–1,02	–2,55	
Anterior minimumthickness pointdeviation [mm]	0,839 [0,438–2,171]	1,050[0,558–2,051]	1,084[0,527–3,107]	0,391[0,233–0,549]	0.001
(ES)	-	–0,71	–0,69	1,72	
Posterior minimumthickness pointdeviation [mm]	0,771[0,375–2,059]	0,953[0,536–1,924]	0,996[0,444–2,941]	0,358[0,197–0,519]	0.000
(ES)	-	–0,65	–0,67	1,66	

Sample was stratified according to the Amsler– Krumeich grading system according to the severity of the disease.

1 Comparing each stage of the disease versus normal eyes, effects size was estimated as follows: 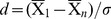, calculating 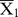 for the normal eyes group, 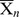 for each stage of the keratoconus group, and 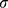 being a pooled standard deviation of compared data.

**Figure 7:**
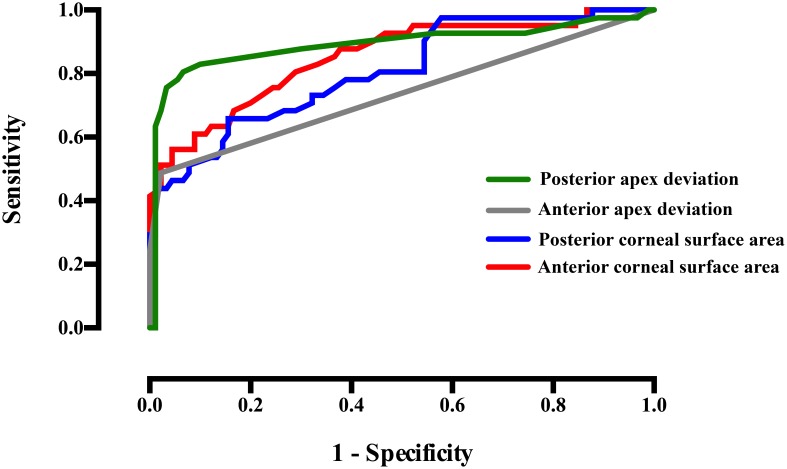
ROC curve modeling the sensitivity versus 1-specificity for variables diagnosing the existence of keratoconus disease (plotted only selected variables with area under the curve over 0.7).

The predictive value of the variables modeled was established through a ROC analysis (see Table 4). Four variables with an area under the curve of over 0.7 were found (see figure 7): anterior corneal surface area (area: 0.847, p<0.000, std. error: 0.038, 95% CI: 0.777 to 0.925), the cut-off value obtained was 43.11 mm^2^, with an associated sensitivity and specificity of 89% and 57% respectively; posterior corneal surface area (area: 0.807, p<0.000, std. error: 0.042, 95% CI: 0,726 to 0,889), the cut-off value obtained was 44.2 mm^2^, with an associated sensitivity and specificity of 91% and 44% respectively; anterior apex deviation (area: 0.735, p<0.000, std. error: 0.053, 95% CI: 0.630 to 0.840), the cut-off value obtained was 0.0015 mm, with an associated sensitivity and specificity of 71.4% and 91.6% respectively; finally, posterior apex deviation (area: 0.891, p<0.000, std. error: 0.039, 95% CI: 0.8146 to 0.9672), the cut-off value obtained was 0.0855 mm, with an associated sensitivity and specificity of 91.1% and 72.9% respectively.

**Table 4:** ROC analysis of sensitivity versus 1-specificity in the disease diagnosis for proposed measurements.

Measurement	Area	Accuracy ratio	p
Total corneal volume [mm^3^]	0.153	–0.694	1.000
Anterior corneal surface area [mm^2^]	0.847	0.694	0.000
Posterior corneal surface area [mm^2^]	0.807	0.614	0.000
Total corneal surface area [mm^2^]	0.430	–0.14	0.171
Sagittal plane apex area [mm^2^]	0.135	–0.73	0.000
Sagittal Plane Area in minimum thickness points [mm^2^]	0.139	–0.722	0.000
Anterior apex deviation [mm]	0.735	0.47	0.000
Posterior apex deviation [mm]	0.891	0.782	0.000
Center of mass X [mm]	0.444	–0.112	0.168
Center of mass Y [mm]	0.485	–0.03	0.911
Net deviation from center of mass XY [mm]	0.503	0.006	0.821
Center of mass Z [mm]	0.567	0.134	0.159
Anterior minimum thickness point deviation [mm]	0.632	0.264	0.010
Posterior minimun thickness point deviation [mm]	0.634	0.268	0.009

## Discussion

This paper proposes a procedure for 3D geometric reconstruction of the human cornea aiming to obtain a solid model that represents a personalized and in vivo morphology of both the anterior and posterior corneal surfaces. This method has been applied with success in both healthy and keratoconic corneas. In order to obtain a personalized characterization of the corneal topography several geometric variables are calculated from the 3D model, enabling to establish a new clinical diagnostic criterion for the differentiation of healthy corneas from corneas with keratoconus.

Some studies have proposed novel methods for geometric modeling of human body parts using a 3D point cloud [40], [41], [45]. However, to the authors knowledge there is no information about the development of this procedure in human corneal modeling. Other authors have reconstructed the corneal surface using approximations to a basic geometry (sphere, ellipsoid) plus a residue representative of the irregularities with respect to the base geometry [21]–[22].

However, these procedures are based on the development of the Zernike polynomials, which have problems that have been widely discussed in the literature [30]–[32]. Some authors try to solve this issue using first a coarse adjustment by means of the Zernike polynomial and secondly a fine adjustment based on a lineal combination of radial basis Gaussian functions [33]. However, this method does not properly represent the corneal geometry when it has high irregularity levels due to ectasia related disorders, both on anterior and posterior surfaces. In contrast, the method proposed in this article provides a reliable solid model that reproduces the geometry of both corneal surfaces, both in keratoconic and healthy corneas, with minimum deviation errors for both surfaces (see figure 2).

Furthermore, current corneal topographers only provide a low data density from the posterior corneal surface, which makes it difficult to obtain a full 3D reconstruction of the human cornea [28]–[29]. For this reason, the criteria applied in this study establishes a method of reconstruction of the corneal region from its geometric center (r = 0 mm) to the beginning of the peripheral zone (r = 4 mm). This criterion is based on two main reasons: i) a geometric one, due to a total of 10752 spatial points representing the geometry of both anterior and posterior corneal surfaces obtained from the SIRIUS; and ii) a clinical one, due to this region comprising 97% of keratoconus cases [42].

A second objective of this work is the characterization of the corneal morphology as a new technique for the clinical diagnosis of keratoconus. There are some studies where the keratoconic eye is diagnosed by studying certain intrinsic geometric parameters directly provided by corneal topographers [3], [46]–[47]. However, in this study we propose new geometric variables extracted from a solid model and representative of the corneal geometry that allow the irregularities in the corneal morphology to be characterized as a new diagnostic technique using a non-invasive clinical approach.

In this sense, the variables that achieved the best results in the diagnosis of the disease were anterior corneal surface area (ROC area: 0.847, p<0.000, std. error: 0.038, 95% CI: 0.777 to 0.925), posterior corneal surface area (ROC area: 0.807, p<0.000, std. error: 0.042, 95% CI: 0,726 to 0,889), anterior apex deviation (ROC area: 0.735, p<0.000, std. error: 0.053, 95% CI: 0.630 to 0.840) and posterior apex deviation (ROC area: 0.891, p<0.000, std. error: 0.039, 95% CI: 0.8146 to 0.9672). Nevertheless, there are other relevant statistical differences between healthy and diseased eyes, and most of variables studied differ between groups (as seen in table 1), making it possible to differentiate healthy corneas from those patients diagnosed with keratoconus. Thus, geometric modeling enables accurate characterization of the human cornea. In addition, from a clinical point of view, the procedure described has established a new approach for the study of eye-related diseases.
